# Modern Urology
*Edited by Hugh Cabot, M.D., F.R.C.S. Published by Lea and Febiger, Philadelphia and New York (706 Sansom Street, Pa.). Price $14. 2 Vols.


**Published:** 1920-07-10

**Authors:** 


### Modern Urology.*

We are in receipt of two most valuable volumes on the subject, edited by Hugh Cabot, M.D., F.R.A.S., Chief of the Genito-Urinary Department of the Massachusetts General Hospital, and containing a series of original contributions by various American authors. One point of particular interest in this work is that in America it is only during the present generation that certain medical men of eminence have devoted themselves exclusively to the subject of urology, and in that all of the contributors to these volumes can claim considerable distinction and experience in general surgery, it is obvious that the effect of the wider views they hold is particularly noticeable in most of the pages. At the same time, each has brought his general knowledge practically to bear upon the minute elaborations of purely urological work, with the result that the reader, though he may not in every case agree with the opinions expressed, finds highly interesting theories propounded and ideas put forward in just that manner which helps his own inventive powers forward. As the editor, in his preface, remarks, such a work as this is in fact a correlated series of monographs and makes up in vigour what it may lack in detail. Illustrations of the best kind and excellent diagrams are provided in great profusion. The general form and production of these volumes are up to the best American standard, which is saying a good deal, and we can confidently recommend "Modern Urology" as a work which should find its place on the book-shelves of every surgeon directly or indirectly interested in this subject.

* Edited by Hugh Cabot, M.D., F.R.C.S. Published by Lea and Febiger, Philadelphia and New York (706 Sansom Street, Pa.). Price $14. 2 Vols.

